# High Frequency of Detection of NDM-Producing Enterobacterales Among Companion Animals Hospitalized in an Italian Veterinary Teaching Hospital

**DOI:** 10.1155/tbed/2622185

**Published:** 2025-01-09

**Authors:** R. Scarpellini, M. Pulido-Vadillo, C. Serna, B. Gonzalez-Zorn, J. L. Blanco, J. F. Delgado-Blas, M. Giunti, S. Piva

**Affiliations:** ^1^Department of Veterinary Sciences, University of Bologna, Ozzano dell'Emilia (BO), Italy; ^2^Antimicrobial Resistance Unit (ARU), Department of Animal Health and VISAVET Health Surveillance Center, Faculty of Veterinary Medicine, Complutense University of Madrid, Madrid, Spain; ^3^Department of Animal Health, Faculty of Veterinary Medicine, Complutense University of Madrid, Madrid, Spain; ^4^Faculty of Veterinary Medicine, Veterinary Teaching Hospital, Complutense University of Madrid, Madrid, Spain; ^5^Biodiversity and Epidemiology of Bacterial Pathogens, Pasteur Institute, Paris City University, Paris, France

## Abstract

Carbapenems are considered one of the most important last-resort classes of antibiotics, and the spread of carbapenem-resistant Enterobacterales (CRE) is a serious concern worldwide. From a One Health point of view, reports on CRE in companion animals are increasing, requiring attention regarding their role in maintenance and direct transmission to humans. The aim of this study was to assess the frequency of detection at admission and the in-hospital acquisition of CRE from perirectal swabs in dogs and cats hospitalized in an Italian Veterinary Teaching Hospital (VTH). Of the 150 patients sampled, 11.3% (*n* = 17) were CRE carriers at admission, 25.6% (*n* = 34) acquired CRE in their commensal microbiota during their hospital stay, and 2% (*n* = 3) developed an infection caused by CRE. Genotypical analysis showed that in 100% (78/78) of the CRE isolates (44 *Escherichia coli*, 33 *Klebsiella pneumoniae*, and 1 *Klebsiella aerogenes*) carbapenem resistance was conferred by the carbapenemase gene bla_NDM_, suggesting an endemic presence of such gene within the hospital. Co-occurrent *β*-lactamase-encoding genes were found in most of the isolates. Risk factors associated with CRE acquisition were length of hospitalization (*p*=0.0002) and treatment with piperacillin–tazobactam (PTZ; *p*=0.0380), indicating potential cross-selection of CRE. These results reinforce the suspicion that companion animals could silently contribute to the maintenance and dissemination of CRE in the local community, posing a threat to global health.

## 1. Introduction

Carbapenems are a class of broad-spectrum *β*-lactam antibiotics, often used to treat severe infections. The emergence of antimicrobial-resistant organisms (AMROs) able to produce extended-spectrum *β*-lactamases (ESBLs) or AmpC *β*-lactamases, such as Enterobacterales like *Escherichia coli* and *Klebsiella pneumoniae*, has made that carbapenems quickly become the last resort antibiotics in hospitals, and their use is typically preserved to treat critical infections caused by such AMROs [1]. However, as antimicrobial resistance increases, their efficacy is threatened by the onset of carbapenem-resistant Enterobacterales (CRE) [1, 2]. CRE have been included in the list 1 “Priority pathogens” (“Critical”), redacted by the WHO in 2017 and recently updated [3], indicated as one of the most important AMROs for which new antibiotics are urgently needed. Acquired carbapenem-resistance in Enterobacterales is typically mediated by the production of carbapenemases, enzymes that hydrolyze carbapenems. Carbapenemases are classified in three different Amber classes [4]: (i) Class A, including the IMI/NMC, KPC, SFC, and GES type enzymes; (ii) Class B, including the IMP, VIM, and the New Dehli-metallo-*β*-lactamases (NDMs) type enzymes; and (iii) Class D, the OXA-48-like type enzymes. Resistance to carbapenems is frequently associated with multidrug resistance (MDR) [2], and specifically with resistance to all *β*-lactams (e.g., third-generation cephalosporins or penicillins combined with *β*-lactamases inhibitors). Carbapenemase production is encoded by antimicrobial resistance genes (ARGs) usually located in plasmids, that can rapidly accumulate and spread several ARGs through horizontal transmission [5, 6].

In veterinary medicine, the regulation of carbapenems varies worldwide, but their use is normally preserved for human medicine. In the EU, they belong to the category A (“Avoid”) classification redacted by the European Medicine Agency (not authorized except for specific clinical circumstances), followed by the 2022/1255 UE regulation that officially banned them from veterinary use. Nevertheless, studies on CRE in companion animals are being increasingly described [2]. This aspect represents a concern from two points of view: first, an infection caused by CRE in companion animals is more difficult to treat because of the limitations on antibiotic use, with a subsequent higher risk of therapeutic failure and second, there is a public health concern in relation to the dissemination of carbapenemase genes and the potential role of pets as reservoirs, especially due to the high frequency of direct contact with humans, with a potential zoonotic transmission risk. The reasons behind the diffusion of CRE in dogs and cats remain understudied, and there are no global surveillance protocols currently in place for companion animals. Remarkably, large healthcare facilities such as Veterinary Teaching Hospitals (VTHs) represent a high-risk place, due to the high number of patients at high risk (e.g., immunocompromised animals) and the wide human–animal interface. Considering that colonization usually precedes infection (the estimation for CRE infections in human medicine suggests that the average time from colonization to infection is 21 days [7]), an active surveillance program capable of screening and estimating the rate of CRE carriers and newly CRE-colonized patients during the hospitalization could be an effective tool for obtaining information and controlling the onset of life-threatening infections. This study aimed to: (i) obtain and analyze data of patients hospitalized at an Italian VTH that were CRE carriers at admission, and those that acquired CRE during the hospital stay; (ii) evaluate the rate of CRE infections in the same patients; and (iii) identify the risk factors associated with CRE carriage and acquisition.

## 2. Materials and Methods

### 2.1. Study Design

A perspective, cross-sectional, and observational study was conducted in the Bologna VTH as a part of a larger surveillance program from May 2021 to May 2023. The study was executed in sessions, performed every 4 months (pulsed surveillance). According with clinical staff's indications, every patient expected to be hospitalized for more than 48 h was sampled at admission and before discharge or death in each session, until reaching 25 patients per session.

### 2.2. Data Collection

For every patient, anamnestic data (species, sex, age, previous antibiotic use in the past 90 days, and previous hospitalization/surgery in the past 90 days) and hospitalization data (length of hospitalization, antibiotic treatment, use of corticosteroids and analgesics, hospitalization in intensive care unit, surgery, anesthesia, and use of invasive devices) were recorded.

### 2.3. Sampling

Perirectal swabs were collected from the same patient at admission (within 12 h) and on the day of discharge/death using sterile swabs with Amies transport medium. Samplings were performed by gently putting the swab in the perirectal area and rotating it for 10–15 s. Samples were stored at 4°C for a maximum of 24 h before being processed.

### 2.4. Isolation and Identification

Every sample was cultured by streaking on selective media for carbapenem-resistant (CR) gram-negative bacteria, CHROMAGAR KPC (Chromagar, Paris, France). After 24–48 h of incubation at 37 ± 1°C under aerobic conditions, chromogenically distinguishable isolated colonies from positive cultures were subcultured on tryptone-soy agar (Oxoid, Wesel, Germany), and subsequently identified by MALDI-TOF mass spectrometry using a Bruker Daltonics MBT SMART equipment (Bruker Daltonics, Germany), following manufacturer' instructions. Colonies identified with a score between 1.5 and 2 were considered only at the genus level.

### 2.5. Clinical Isolates

Clinical isolates from patients with a positive bacteriological culture during the hospital stay were registered as part of the normal routine diagnostics. Among them, Enterobacterales isolates that showed phenotypic resistance to piperacillin–tazobactam (PTZ) 110 μg, according to CLSI breakpoints [8], were considered as a suspected CRE infection. The identification was carried out as previously described.

### 2.6. Antimicrobial Susceptibility Testing

Isolates (including clinical isolates from suspected CRE infections) were screened for confirmation of the phenotypical resistance to carbapenems through the disc diffusion test (DDT). Following EUCAST guidelines [9], a screening to confirm carbapenem resistance was performed by DDT against ertapenem 10 μg (zone diameter < 25 mm).

### 2.7. ARG Detection

Single and multiplex PCRs were performed to assess the presence of resistance genes. Isolates identified as Enterobacterales were tested for the presence of several ESBL genes (bla_CTX-M_ group 1, bla_SHV_, bla_OXA−1_-like, and bla_TEM_), carbapenemase genes (bla_KPC−1_-bla_KPC−5_, bla_VIM_, bla_IMP_, and bla_OXA−48_), and various AmpC genes (bla_CMY−2_-bla_CMY−7_, bla_CMY−12_-bla_CMY−18_, bla_CMY−21_-bla_CMY 23_, bla_LAT−1_-bla_LAT−3_, and bla_BIL−1_) following the protocols described by Dallenne et al. [10]; for the detection of bla_CTX-M_, the primers described by Kirasitin et al. [11] were used. In addition, the protocol described by Poirel et al. [12] was used to assess the presence of bla_NDM_. Clinical isolates from suspected CRE infections were tested only for the presence of bla_NDM_. The list of positive controls used is present in Table S1.

### 2.8. Descriptive Statistical Analyses

Carriage at admission was determined in patients with a positive sample at admission. In-hospital acquisition was determined in animals that were negative at the admission sampling and positive at discharge. Patients with a positive sample at admission were not included in the evaluation of the in-hospital acquisition. A temporal trend description of the percentage of patients carriers at admission, the in-hospital acquisition and CRE infections cases among the different sampling sessions was also performed. Risk factors analysis was conducted. The association between carriage at admission and the anamnestic data was assessed using univariable logistic regression, and variables with a *p*  < 0.05 were included in the multivariable logistic model built using stepwise selection at *p*  < 0.05. Normality and heteroskedasticity of data were corrected with the Shapiro–Wilk test and the Levene's test. The same analysis was used to assess the association between the anamnestic/hospitalization data and the in-hospital acquisition. Data were checked for multicollinearity with the Belsley–Kuh–Welsch technique. Statistical analyses were performed with MedCalc (version v22.009).

## 3. Results

### 3.1. Anamnestic and Hospitalization Data

A total of 150 patients were sampled in six sessions. One-hundred and five out of 150 (70%, 56 males and 49 females) were dogs, while 45 out of 150 (30%, 36 males and 9 females) were cats. Average age was 9.1 years (95% CI: 8.5–9.9), while the average length of hospitalization was 5.3 days (95% CI: 4.6–6.1). Distribution of hospitalization data is shown in Table 1. Considering antibiotic use up to 90 days prior to hospitalization (Table 2), amoxicillin–clavulanate (AMC) was the most frequent drug, used in 16 out of 150 patients (10.7%), followed by marbofloxacin (MRB), enrofloxacin (ENR), and ampicillin–sulbactam (AMS), used all three in 6/150 (4%) patients. Antibiotic treatment during hospitalization was performed in 70.7% of patients (106/150). AMC was the most frequent antibiotic (57/150 patients, 38%), followed by MRB (23/150 patients, 15.3%), first generation cephalosporins (17/150 patients, 11.3%), and PTZ (15/150 patients, 10%). Three patients out of 150 (2%, 3 dogs) developed a suspected CRE infection during their stay. Seventeen out of 150 patients (11.3%, 95% CI: 4.1–18.5; 13 dogs and 4 cats) were detected as CRE carriers at admission, and all (17/17) were sill CRE carriers at discharge, while 34 out of 133 (25.6%, 95% CI: 17.6–33.6; 23 dogs and 11 cats) acquired CRE during hospitalization.

### 3.2. Bacterial Identificationand Phenotypic/Genotypic Profiles

A total of 80 CRE commensal isolates were identified, of which 78 (97.5%) were confirmed to be resistant to ertapenem by DDT. The most common isolated species was *E. coli* (*n* = 44), followed by *K. pneumoniae* (*n* = 33) and *Klebsiella aerogenes* (*n* = 1). Genotypical analysis (Table 3 and Figure S1) showed the presence of the bla_NDM_ gene in 100% of the isolates (78/78), while bla_VIM_, bla_IMP_, bla_OXA−48_-like, and bla_KPC−1_-bla_KPC−5_ genes were not found. The ESBL encoding genes, bla_OXA−1_-like, bla_TEM_, bla_CTX-M_ group 1, and bla_SHV_ were found in 82.9% (63/76), 78.9% (60/76), 76.6% (59/77), and 43.4% (33/76) of tested isolates, respectively. The AmpC genes (bla_CMY−2_-bla_CMY−7_, bla_CMY−12_-bla_CMY−18_, bla_CMY−21_-bla_CMY 23_, bla_LAT−1_-bla_LAT−3_, and bla_BIL−1_) were found in 21/78 of isolates (26.9%). The copresence of bla_NDM_ and at least one of the other detected genes investigated was confirmed in 67/75 isolates (89.3%), while 31 isolates (39.7%) harbored simultaneously bla_NDM_ and all tested ESBL genes (bla_OXA−1_-like, bla_TEM_, bla_CTX-M_ group 1, and bla_SHV_), including 5 (6.4%) that also tested positive for the AmpC genes. Additionally, three clinical isolates from suspected CRE infections (two *K. pneumoniae* and one *E. coli*) were registered. All these isolates were found in dogs, one from a surgical site infection and two from abdominal effusions. Two patients in which such isolates were found were CRE carriers at admission, while one acquired CRE during hospitalization. The bla_NDM_ gene was detected in all the three isolates (100%). All the three patients died during the hospital stay.

### 3.3. Temporal Trend

**Figure 1:**
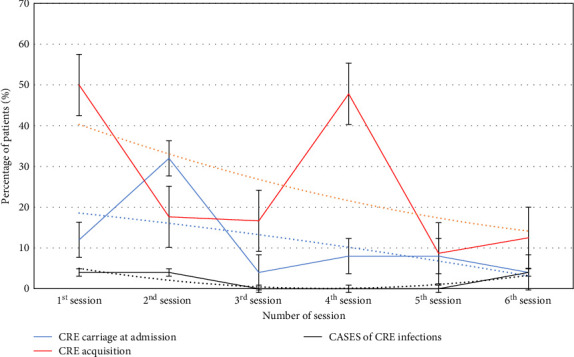
Temporal trend of carbapenem-resistant Enterobacterales (CRE) carriage at admission, CRE acquisition and CRE infection cases among the different sampling sessions, from May 2021 (1^st^ session) to May 2023 (6^th^ session). Order 2 polynomial tendency lines are also shown for each group.

The percentage of patients screened among the six sessions found to be CRE carriers at admission and that acquired CRE during hospitalization is shown in Figure 1, as well as the percentage of patients that developed a CRE infection. Notably, a carriage peak at admission was detected in the second session, while the rate of acquisition peaked in the fourth session.

### 3.4. Risk Factor Analysis

In the multivariate analysis, none of the variables considered was associated with a higher chance for CRE carriage at admission. Considering in-hospital acquisition, the multivariate analysis showed that the length of hospitalization (*p*=0.0027) and the treatment with PTZ (*p*=0.0380) were significantly associated with CRE acquisition.

## 4. Discussion

The percentages of CRE detected in our study, both regarding carriage at admission and in-hospital acquisition, are considerably higher compared with the few other studies in Europe [2, 13–15] and are concerning especially from a One Health perspective, indicating companion animals as an overlooked source of CRE, potentially acting as reservoirs. Although the use of carbapenems in pets in Italy was first restricted and then banned, 11.3% of hospitalized patients carried CRE in their gut microbiota at admission, and 25.6% of patients who were negatives at admission acquired CRE during their hospital stay. The high detection frequency at admission could be partially explained by the fact that, although no significant risk factors were detected, more than half of patients carriers at admission (9/17) was hospitalized at the same VTH in the previous 90 days, where they possibly acquired CRE, subsequently detected when they returned to the VTH. Furthermore, considering that the samplings were executed with a turnover time from the admission of maximum 12 h, the possibility that a patient became colonized by CRE in such timeframe, with a consequent overestimation of CRE carriage at admission (and underestimation of CRE in-hospital acquisition) must be considered. The high acquisition rates suggest that carbapenemase genes could have been massively acquired through horizontal transmission, or that commensal, and nonpathogenic CRE could have colonized the patients' gut flora, even in the absence of clinical infections. Indeed, three of the patients investigated (2%) developed a CRE infection, with no temporal correspondence with the percentage of patients that acquired CRE in the hospital (Figure 1), confirming that CRE colonization could silently spread within a healthcare setting. Nevertheless, the three clinical cases of CRE infection represent the evidence of the problem, with a fatal outcome due to the inability to treat such infections in veterinary practice. Such findings must remark on the importance of surveillance and infection control policies also in small animal medicine, where there are fewer therapeutic options.

CR was conferred by the bla_NDM_ gene in all the isolates, strongly suggesting the endemic presence of such ARG, selected and maintained within the VTH. NDMs are one of the most concerning carbapenemase types, and their emergence in small animal practice is increasingly being described worldwide [16–23], including Italy, where the presence of bla_NDM−5_ was first described by Alba et al. [24] in 2021 in an *E. coli* isolate from a dog urine sample in Northern Italy. In the Bologna province, NDMs have been reported in a 2-year study on rectal swabs from hospitalized human patients, but in a considerably lower proportion (0.25% of total swabs and 6.2% of total CRE isolates) [25]. On a National level, the 2022 report from the Italian National Institute of Health showed that NDMs were found in approximately 8% of total CRE isolated from bacteremia cases [26]. In our case, the origin of such a large CRE spread remains unclear, stated that carbapenems were not used during the study period and could be the driving force of their selection and maintenance. As hypothesized by Sellera, Da Silva, and Lincopan [27], there are two main vias for companion animals to acquire CRE, through contact with a colonized human host or through a contaminated environment. In our case, the possibility of a primary interspecies transmission from humans (reverse zoonosis) should be considered; additionally, since animals had no direct contact during their stay, contamination through a common source, aided by selective pressure exerted by antibiotic use, could have facilitated the permanence of CRE in the VTH, with a subsequent clonal and/or plasmidic spread over time. This transmission from the environment to animals may be direct or through hospital personnel. A similar Swiss study by Nigg et al. [28], reported the acquisition of clonal CR *E. coli* harboring bla_OXA−181_ and bla_CMY−42_ by patients after hospitalization (21.6%), although only one dog was found to be positive at admission, suggesting a within-hospital spread of carbapenemase-encoding plasmids. On the other hand, bla_CTX-M_ group 1, bla_OXA−1_-like, and bla_TEM_ were confirmed to be the most common ESBL genes in pets in Europe [29]. The fact that 39.7% of CRE isolates harbored all the four ESBL genes investigated and bla_NDM_ (and in 6.4% of the cases, AmpC genes) highlights that Enterobacterales in pets are able to accumulate a vast repertoire of ARGs over time, which often confer a higher MDR rates (up to 95%, according to other studies) and resistance diversity than in human isolates [30, 31].

According to the risk factor analysis of CRE in-hospital acquisition, the length of hospitalization appeared to be a relevant factor for CRE colonization. This result aligns with the literature on AMROs in-hospital colonization [32–36] and points at hospital stay reduction as a critical measure in order to minimize the chance of AMROs colonization. However, this indication could be more challenging for small animal practice, because the “home treatment” is more complex to achieve correctly, but it still must be considered to reduce the risks. In a study conducted by Lavigne et al. [37], risk factors analysis for NDM-5 producing *E. coli* in-hospital acquisition identified that exposure to the anesthesia service, surgery service, and endotracheal intubation were significantly associated with higher chances of acquisition. In our case, such factors were included in the model but did not entail a risk for CRE acquisition. Patients treated with PTZ during their hospital stay (until its ban from veterinary use in the EU with the 2022/1255 regulation) were statistically associated with a higher chance of acquiring CRE, suggesting cross-selection for CRE caused by the use of PTZ. Specifically, PTZ, a very effective antibiotic against ESBL-producing Enterobacterales, has been reported to select for changes associated with MDR [38], and its long-term use could have favored the selection of bla_NDM_ within the VTH. Studies from human medicine [39–41] have reported that PTZ resistance could be mediated by the expression of bla_OXA−1_ or through a combination or overexpression of other genes such as bla_TEM_, bla_CTX-M−15_, and bla_CMY−2_. The high prevalence of these genes in copresence with bla_NDM_ reinforces the hypothesis of a cross-selection. Notably, cases of patients that acquired CRE within the hospital were registered even after the official ban of PTZ in February 2023, suggesting that the presence of bla_NDM_ resence in the hospital could have been maintained in the long term.

Screening patients at admission is a useful tool that provides data on AMR spread in pets and allows better management of patients. Indeed, if a positive carrier is rapidly detected, additional preventive measures, such as contact isolation, can be implemented. The European Society of Clinical Microbiology and Infectious Diseases (ESCMID) guidelines strongly recommend the implementation of extra preventive measures in such cases [42]. Furthermore, the communication of the screening results to hospital personnel should imply an increase in attention and compliance with good hygienic practices, such as hand washing. Nevertheless, the effectiveness of such precautions to reduce in-hospital transmission and healthcare-associated infections remains a matter of debate [43, 44]. The main complication regards the turnaround times, often too long to allow proper risk reduction [45]. Furthermore, veterinary healthcare facilities often lack the workload and resources to execute universal contact isolation for all positive patients screened at admission, especially in zones with high endemic rates. In a study by Kaspar et al. [46] from human medicine more than 80% of patients who were transferred to Swiss hospitals from high-risk areas were unnecessarily screened for AMROs; they recommended instead more limited screening based on identifiable risk factors. Risk-based admission screening for CRE carriers that focuses only on patients with a major risk according to their clinical history (e.g., by developing an individual “risk score”) could reduce the costs compared with an extensive and indiscriminate screening of all patients.

Sampling patients at discharge allows to evaluate the in-hospital acquisition and associated risk factors. This can help to detect potential epidemic transmissions before the onset of infections and to limit the spread of AMROs outside the hospital. The timely detection of patients that acquire CRE, with a subsequent follow-up over time, might be relevant to avoid the spread of this concerning resistance in the local community, especially in relation to their importance for public health. In Enterobacterales, the possibility of sharing resistance between humans and pets has already been demonstrated [18, 31, 47], highlighting the potential for interspecies transmission. In a similar study from Switzerland by Nigg et al. [28], dogs positive for CRE at discharge were still carriers after 4 months, but were negative after 6 months. In our case, we did not conduct follow-up studies, but we can assume a similar duration of AMR persistence, although studies from human medicine have estimated durations of CR colonization up to 12 months [48].

This study aimed to describe a program of active screening surveillance for CRE detection in companion animals hospitalized for more than 48 h at an Italian VTH. It is well established that patients become colonized before developing an infectious condition [49]; therefore, monitoring CRE presence in the patients' microbiota can be a useful indicator for a general evaluation of their prevalence, possibly allowing to anticipate the onset of CRE infections. This consideration is particularly important in large, tertiary care facilities such as VTHs, that often possess multiple services and areas, involving a high number of personnel. In our case, the VTH of this study is a large and multispecies hospital, with 16 different services operating for small animals and three for horses, and an average of 80 patients hospitalized per month. In these facilities, a surveillance capable of preventing and limiting the spread of CRE and related ARGs within and outside the hospital is crucial. The choice to perform this type of surveillance in pulsed sessions (every 4 months, until reaching 25 patients in each session), was in line with the guidelines for Infection Control by Anderson, Wimmers and Weese [50] and was mainly due to the impossibility to afford the costs, both timely and economically related, derived from an extensive and continuous active screening. Pulsed surveillance can optimize such costs, still furnishing a detailed evaluation in determined timeframes.

This study has several limitations. First, although the way in which it was performed over time (“pulsed”) allowed to reduce the costs and to obtain a general overview about the endemic situation, it did not consent to properly evaluate the real, continuous trend over time, especially about in-hospital acquisition. Second, although genotypical confirmatory tests were performed for the most common resistance genes, a deeper epidemiological and genotypical investigation could enable a more comprehensive understanding of the bacterial and genetic dynamics. Third, the number of patients enrolled (*n* = 150) may have led to a failure to detect significant risk factors. Fourth, as previously mentioned, follow-up screenings to determine how long patients continued to be CRE carriers after hospitalization were not performed.

## 5. Conclusions

CRE prevalence seems to be increasing in small animal practice and it has been increasingly described. This study highlights the potential role of VTHs in the selection, maintenance, and spread of CRE through patients' gut microbiota even in the absence of carbapenems use, indicating how companion animals could silently contribute to AMR dissemination into the local community. Specifically, the use of antibiotics such as PTZ could have led to the cross-selection of highly important ARGs such as bla_NDM_. Considering that CRE are one of the most concerning public health problems, active surveillance programs should be implemented also in veterinary settings. Additionally, the integration of data from animals with local human medical settings should be considered.

## Figures and Tables

**Table 1:** Hospitalization data of the 150 patients (dogs and cats) included in the study during six different sessions, from May 2021 to May 2023.

Hospitalization data	Number of patients	%
Antimicrobial treatment in the previous 90 days	44	29.3
Antimicrobial treatment with more than one drug in the previous 90 days	9	6.6
Hospitalization/surgery in the previous 90 days	35	23.3
Antimicrobial treatment during the hospitalization	106	70.7
Antimicrobial treatment with more than one drug during the hospitalization	22	14.7
Opioid analgesics use during the hospitalization	93	62
Treatment with corticosteroids during the hospitalization	30	20
Hospitalization in intensive care unit	100	66.7
Surgery during the hospitalization	59	39.3
Anesthesia room	73	48.7
Use of urinary catheter	20	13.3
Use of nasogastric tube	34	22.7
Use of other invasive devices (central venous catheter, surgical drainage tube, ureteral stent)	25	16.7

**Table 2:** Antibiotic use (prior and during hospitalization) in the 150 patients (dogs and cats) included in the study during six different sessions, from May 2021 to May 2023.

Antibiotic drug	Number of patients treated up to 90 days prior to hospitalization	%	Number of patients treated during hospitalization	%
AK	0	0.0	3	2.0
AMC	16	10.7	10	6.7
AMS	6	4.0	57	38.0
DOXI	4	2.7	7	4.7
ENR	6	4.0	0	0.0
1^st^ generation cephalosporins	5	3.3	17	11.3
3^rd^ generation cephalosporins	2	1.3	0	0.0
MRB	6	4.0	23	15.3
Others/unknown	8	5.3	3	2.0
PTZ	1	0.7	15	10.0

Abbreviations: AK, amikacin; AMC, amoxicillin–clavulanate; AMS, ampicillin–sulbactam; DOXI, doxycycline; ENR, enrofloxacin; MRB, marbofloxacin; PTZ, piperacillin–tazobactam.

**Table 3:** Genotypic resistance profiles regarding the bla genes of the 78 CRE isolates from perineal swabs of dogs and cats hospitalized in the VTH during the study.

*β*-Lactamase(s) targeted	Resistance gene tested	Total isolates tested	Number of positive isolates	Prevalence (%)
VIM variants including VIM-1 and VIM-2	bla_VIM_	78	0	0
IMP variants except IMP-9, IMP-16, IMP-18, IMP-22 and IMP-25	bla_IMP_	78	0	0
OXA-48-like	bla_OXA−48_-like	78	0	0
KPC-1 to KPC-5	bla_KPC−1_-bla_KPC−5_	78	0	0
NDM	bla_NDM_	78	78	100
TEM variants including TEM-1 and TEM-2	bla_TEM_	76	60	78.9
CTX-M	bla_CTX-M_ group 1	77	59	76.6
SHV variants including SHV-1	bla_SHV_	76	33	43.4
OXA-1, OXA-4, and OXA-30	bla_OXA−1_-like	76	63	82.9
LAT-1 to LAT-3, BIL-1, CMY-2 to CMY-7, CMY-12 to CMY-18 and CMY-21 to CMY-23	bla_CMY−2_-bla_CMY−7_, bla_CMY−12_-bla_CMY−18_, bla_CMY−21_-bla_CMY 23_, bla_LAT−1_-bla_LAT−3_, bla_BIL−1_	78	21	26.9

Abbreviations: CRE, carbapenem-resistant Enterobacterales; NDM, New Dehli-metallo-*β*-lactamase; VTH, Veterinary Teaching Hospital.

## Data Availability

The data that support the findings of this study are available from the corresponding author upon reasonable request.

## Supporting Information

Supporting Information Table S1: provides the list of positive controls used, while Figure S1: shows an image of the PCR results.
