# Supplementary material for: High Frequency of Detection of NDM-Producing Enterobacterales Among Companion Animals Hospitalized in an Italian Veterinary Teaching Hospital
Source: Transbound Emerg Dis. 2025 Jan 9;2025:2622185. doi: 10.1155/tbed/2622185 (PMC12017035; doi:10.1155/tbed/2622185)
Supplement: Supporting Information — Table S1: provides the list of positive controls used, while Figure S1: shows an image of the PCR results. [file 2622185.f1.docx]

**Supplementary Material**

**TABLE S1. List of positive controls of the study.**

| **Gene tested** | **Positive control** | **Reference** |
| --- | --- | --- |
| ***bla*_KPC 1-5_** | ***Klebsiella pneumoniae* ST258 *bla*_KPC-3_** | **[1]** |
| ***bla*_VIM_** | **pMBA::*bla*_VIM-2_ plasmid** | **[2]** |
| ***bla*_IMP_** | **pMBA::*bla*_IMP-2_ plasmid** | **[2]** |
| ***bla*_OXA-48_** | ***Escherichia coli* MDS42 :: pOXA-48** | **Personal collection of the Antimicrobial Resistance Unit** |
| ***bla* _NDM_** | ***E. coli* ST1196 BB1471 pCW-NDM-1** | **[3]** |
| ***bla*_CTX-M_ group 1** | ***K.pneumoniae* ST307** | **Personal collection of the Laboratory of Veterinary Bacteriology** |
| ***bla*_SHV,_** | ***K.pneumoniae* ST307** | **Personal collection of the Laboratory of Veterinary Bacteriology** |
| ***bla*_OXA-1_-like** | ***K.pneumoniae* ST307** | **Personal collection of the Laboratory of Veterinary Bacteriology** |
| ***bla*_TEM_** | ***K.pneumoniae* ST307** | **Personal collection of the Laboratory of Veterinary Bacteriology** |
| ***bla*_CMY-2_-*bla*_CMY-7,_ *bla*_CMY-12_-*bla*_CMY-18,_ *bla*_CMY-21_-*bla*_CMY 23_*_,_ bla*_LAT-1_-*bla*_LAT-3_*, bla*_BIL-1_** | ***E.coli* ST 167** | **Personal collection of the Laboratory of Veterinary Bacteriology** |

**Figure 1. Image of the PCR results for *bla*_NDM_. Amplicon size: 621 bp [4].**

**
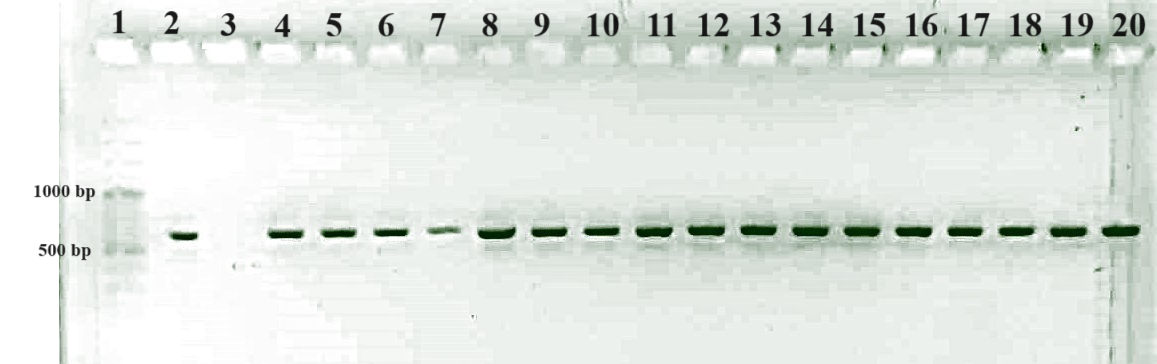
**

1. 100 bp DNA ladder
2. *bla* _NDM_ positive control (*E. coli* ST1196 BB1471 pCW-NDM-1)
3. PCR negative control (water)

4-20) PCR positive isolates

**References**

- [1] A. Cannatelli *et al.*, In vivo emergence of colistin resistance in *Klebsiella pneumoniae* producing KPC-type carbapenemases mediated by insertional inactivation of the PhoQ/PhoP mgrB regulator, *Antimicrobial agents and chemotherapy* 57,11 (2013)
- [2] A. Hipólito, L. García-Pastor, E. Vergara *et al.,* Profile and resistance levels of 136 integron resistance genes, *npj Antimicrob Resist* 1, 13 (2023).
- [3] J.F Delgado-Blas, C.Valenzuela Agüi, E. Marin Rodriguez, C. Serna, N.Montero, C.K.S. Saba, B. Gonzalez-Zorn, Dissemination Routes of Carbapenem and Pan-Aminoglycoside Resistance Mechanisms in Hospital and Urban Wastewater Canalizations of Ghana. *mSystems* 22 (2022). doi: 10.1128/msystems.01019-21.
- [4] L. Poirel, T.R. Walsh, V. Cuvillier, P. Nordmann, Multiplex PCR for detection of acquired carbapenemase genes, Diagnostic Microbiology and Infectious Disease 70 (2011) 119–123. https://doi.org/10.1016/j.diagmicrobio.2010.12.002.
